# Antibiotic misuse among children with diarrhea in China: results from a national survey

**DOI:** 10.7717/peerj.2668

**Published:** 2016-11-03

**Authors:** Ruili Li, Feng Xiao, Xiaoguo Zheng, Huimin Yang, Lihong Wang, Delu Yin, Tao Yin, Qianqian Xin, Bowen Chen

**Affiliations:** Children Health and Development Department, Capital Institute of Paediatrics, Beijing, China

**Keywords:** Self-medication, Antibiotic misuse, Children, Diarrhea, China

## Abstract

**Background:**

Antibiotic resistance is one of the world’s biggest public health issues, and the situation in China is particularly grave. The objective of this study is to investigate the antibiotics usage pattern among Chinese children and provide further insight in developing strategies for promoting public health education.

**Methods:**

This is a cross-sectional study, in the study, participants are from 53,665 guardians of children aged 0–6 years, who were recruited with multistage stratified random cluster sampling in 2013/2014 from 46 community health centers in 14 provinces across China Mainland. Children’s guardians completed surveys on their previous experience on using antibiotics in treating diarrhea of their children without a prescription from any pediatrician. Odds ratios (ORs) and 95% confidential intervals (CIs) for the association between antibiotic use and its predictors were estimated using multilevel logistic regression models, with antibiotic rational use group as a reference group.

**Results:**

The prevalence of antibiotic misuse among children with diarrhea in the eastern, middle and western areas of China and associations between antibiotic misuse and its predictors were studied. The average rate of antibiotic misuse is 35.12%. Multilevel logistic regression revealed that living in urban areas (OR = 0.79 (0.76, 0.83)), female children (OR = 0.92 (0.88, 0.96)), guardians having higher education (OR = 0.60 (0.55, 0.66)), being raised by parents (OR = 0.90 (0.85, 0.94)), guardians having basic health knowledge (OR = 0.82 (0.79, 0.86)) are protective factors and children’s age (1–3 years OR = 1.62 (1.54, 1.71)); 4–6 years OR = 1.90 (1.77, 2.03)) is a risk factor of antibiotic misuse among children aged 0–6 years with diarrhea in China.

**Conclusions:**

Our findings confirmed that there has been a high rate of antibiotic misuse without a prescription in children with diarrhea in China, which requires considerable attention. Suitable regulations and interventions are needed to solve this problem.

## Introduction

The abuse of antibiotics has been identified as an emerging threat to global public health because unnecessary use of antibiotics can result in antibiotic resistance to pathogenic bacteria (Bi, Tong & Parton, 2000; Nyquist et al., 1998; Yu et al., 2014). It is estimated that up to 50% of all antibiotics use is inappropriate (Nyquist et al., 1998). The issue of antibiotic misuse among children is of special concern in low- and middle-income countries, with consideration of poor sanitation in these contexts (Yu et al., 2014).

There are various factors that may influence antibiotic misuse. Findings from many studies have showed that inappropriate use of antibiotics is strongly associated with demographic characteristics, including education level (Hoffmann et al., 2014; Kim, Moon & Kim, 2011; Mouhieddine et al., 2015), family income (Barah & Gonçalves, 2010; Mouhieddine et al., 2015; You et al., 2008), place of residence (Godycki-Cwirko et al., 2014), as well as other factors, such as gender, age and parenting styles of the children (Bi, Tong & Parton, 2000).

Another important factor contributing to antibiotic resistance is self-medication, which refers to guardians who use nonprescription medicines (specifically “antibiotics” in this article) to treat perceived infection of their children themselves without approval or supervision of a medical worker (Du & Knopf, 2009; Pavydė et al., 2015). Self-medication serving as a supplement to or even instead of medical care, has been common in both developed and low-income developing countries (Bi, Tong & Parton, 2000). However, parents and other guardians may have insufficient knowledge of the antibiotics they use, which can lead to drug misuse or overuse among self-medicating children (Costello, Wong & Nunn, 2004; Du & Knopf, 2009). Besides potential antibiotic resistance, the misuse of antibiotics might cover the severity of diseases, obscure diagnosis, and even complicate therapy (Al-Ramahi, 2013; Costello, Wong & Nunn, 2004).

There has been very little research on the prevalence and its determinants of antibiotics misuse among children in China nationally. Due to the great variation in antibiotic misuse across nation, determinants are needed in each country prior to development of any effective intervention programs (Pavydė et al., 2015).

Since late 1970s, oral rehydration solution (ORS) has become the most commonly recommended treatment for diarrhea. It is unnecessary to use antibiotics in acute diarrhea. But parents do not use ORS because it does not treat the symptoms, it tastes bad, or because it does not look like a “real medicine.” Under “One-child” policy in China, a majority of parents worry excessively when their children suffer from the diarrhea. As a result, they prefer to give antibiotics to their children without prescriptions. Against this background, using the data from the Health Management Survey for children aged 0–6 years, we present the prevalence and its determinants of self-medication use among the national child population in China, focusing on infant and preschool children diarrhea, one of the common diseases among children aged 0–6 years.

## Materials and Methods

### Study design and participants

This study was performed based on a cross-sectional survey with a multi-stage stratified cluster sampling method. At the first sampling stage, five provinces of Eastern China (Shanghai, Fujian, Liaoning, Shandong, and Zhejiang), four provinces of Middle China (Anhui, Hubei, Hunan, and Jiangxi) and five provinces of Western China (Sichuan, Shaanxi, Chongqing, Guizhou, and Inner Mongolia) were selected. At the second stage, approximately equal numbers of community health centers in urban and suburban areas were recruited in every province. At the third and last stages, all of the children aged 0–6 years living in the sampled communities were enrolled in our study. The guardians of the children received a paper questionnaire and answered voluntarily these questions after being informed consent. It was conducted in 2013/2014 among 46 community health centers in 14 provinces across China Mainland, with collaborators from Capital Institute of Pediatrics and 46 collaborating local cites. The response rate was 87.6% (53,665/61,279).

### Human ethics

The Ethical approval was obtained from the Ethics Committee on Human Research at Capital Institute of Pediatrics (SHERLLM 2013014).

### Measures

Questionnaires were personally distributed by the researchers and healthcare workers in 46 collaborating local cites and collected on the same day. The content of the questionnaire included information of the children and guardians, guardian having basic health knowledge related to parenting, and guardian’s experience with antibiotic use. All the items of the questionnaire were selected from the national health survey. Before the formal survey, we conducted a pilot study to validate the questionnaire. The reliability and validity fit the requirement.

The guardian’s knowledge of basic health related to parenting was measured with one question: How long should a mother exclusively breastfeed her baby before introducing complementary foods? Participants were marked as (1) “good” at having knowledge of basic health related to parenting if answered “six months,” (2) “bad “at basic health knowledge related to parenting if answered something else.

The participants’ previous experience with antibiotics in infantile diarrhea was evaluated with one question: “How often do you give antibiotics to your child to treat diarrhea without any prescriptions from a pediatrician?” with possible responses of (1) Usually;(2) Occasionally; (3) Never, (4) Not applicable (NA), my child never have diarrhea, and (5) NA, I went to see a doctor when my child had diarrhea. Item 1 and 2 are classified into a category of “Misuse,” item 3 and 5 are classified into a category of “Rational use,” and item 4 is marked as NA.

### Statistical analysis

All analyses were performed using Stata/SE version 13.1 (StataCorp, College Station, TX, USA). Contingency tables were used to summarize the prevalence of demographics and primary outcomes among participants from three areas of China. Pearson chi-squared tests and Cochran-Mantel-Haenszel Chi-Squared tests were used to evaluate the associations among experience with antibiotics and its predictors. Odds ratios (ORs) and 95% confidential intervals (CIs) for the association between antibiotics use and its predictors were estimated using logistic regression models, with antibiotics rational use group as the reference group. We used “melogit” command to fit multilevel logistic regression models for the hierarchical data, in the multilevel models, level 1 variable is child-guardian, level 2 variable is community and level 3 variable is province.

## Results

### Characteristics of children and their guardians

The results for demographic characteristics of children and their guardians are summarized in Table 1. A total of 53,665 questionnaires were completed with 20,392 from **Western China**, 14,955 from **Middle China** and 18,318 from **Eastern China**. Where 28,379 (52.88%) children are boys and 25,286 (47.12%) children are girls. Most of the children (62.36%) lived in urban areas and about half of them aged from 1 to 3 years. Nearly 80% of the children were looked after by their parents. The education level of the guardians varied across the areas, highest in **Eastern China**, followed by **Western China** and then **Middle China**. The 24.21% of the household income per capita is higher than 5,000 RMB. The average monthly income is highest in **Eastern China** and lowest in **Middle China**. About half of the guardians have basic knowledge on parenting.

**Table 1:** Characteristics of the study population, by area^a^.

Characteristics	Western China (n = 20,392)	Middle China (n = 14,955)	Eastern China (n = 18,318)	Total (n = 53,665)
Place of residence, %(n)				
Suburb	47.27 (9,639)	32.72 (4,894)	30.94 (5,668)	37.64 (20,201)
Urban	52.73 (10,753)	67.28 (10,061)	69.06 (12,650)	62.36 (33,464)
Children’s gender, %(n)				
Male	52.83 (10,774)	54.39 (8,134)	51.70 (9,471)	52.88 (28,379)
Female	47.17 (9,618)	45.61 (6,821)	48.30 (8,847)	47.12 (25,286)
Children’s age, %(n)				
0 year	35.60 (7,260)	27.76 (4,152)	28.26 (5,176)	30.91 (16,588)
1–3 years	54.41 (11,095)	50.59 (7,565)	52.69 (9,652)	52.76 (28,312)
4–6 years	9.99 (2,037)	21.65 (3,238)	19.05 (3,490)	16.33 (8,765)
Household income per capita, %(n)				
Low (< 3,000 RMB)	44.50 (8,913)	47.12 (6,924)	37.69 (6,766)	42.91 (22,603)
Middle (3,001–5,000 RMB)	32.08 (6,425)	35.56 (5,225)	31.57 (5,666)	32.87 (17,316)
High (> 5,000 RMB)	23.42 (4,692)	17.31 (2,544)	30.74 (5,518)	24.21 (12,754)
Education of guardians, %(n)				
High school and below	61.59 (12,422)	69.33 (10,300)	57.69 (10,508)	62.41 (33,230)
College and above	38.41 (7,748)	30.67 (4,557)	42.31 (7,706)	37.59 (20,011)
Parenting, %(n)				
Others	20.57 (4,195)	25.28 (3,780)	17.60 (3,224)	20.87 (11,199)
Parents	79.43 (16,197)	74.72 (11,175)	82.40 (15,094)	79.13 (42,466)
Basic health knowledge of parenting, %(n)				
Bad	45.82 (8,970)	52.71 (7,624)	45.47 (8,049)	47.63 (24,643)
Good	54.18 (10,607)	47.29 (6,840)	54.53 (9,651)	52.37 (27,098)

**Note:**

a The children without diarrhea were remarked as missing.

### The prevalence of antibiotic misuse and its predictors

Findings for the prevalence of antibiotic misuse and its predictors are presented in Table 2. The 35.12% of the children have taken antibiotics to treat diarrhea without any prescription, the prevalence is **highest in Middle China** (40.61%), **lowest in Eastern China** (32.98%). Variables associated with antibiotic misuse were regions, children’s gender and age, guardians’ parenting style, income, education, and basic health knowledge.

**Table 2:** The prevalence of antibiotic misuse and its variation.

Predictors	Total (n = 39,224)		P value
	Rational use	Misuse	
Place of residence, %(n)			0.000
Suburb	59.91 (8,645)	40.09 (5,785)	
Urban	67.77 (16,804)	32.23 (7,990)	
Children’s gender, %(n)			0.000
Male	63.96 (13,301)	36.04 (7,495)	
Female	65.92 (12,148)	34.08 (6,280)	
Children’s age, %(n)			0.000
0 year	73.40 (8,016)	26.60 (2,905)	
1–3 years	62.71 (13,628)	37.29 (8,105)	
4–6 years	57.91 (3,805)	42.09 (2,765)	
Household income per capita, %(n)			0.000
Low (< 3,000 RMB)	61.17 (10,172)	38.83 (6,456)	
Middle (3,001–5,000 RMB)	65.84 (8,265)	34.16 (4,289)	
High (> 5,000 RMB)	70.14 (6,693)	29.86 (2,849)	
Education of guardians, %(n)			0.000
High school and below	60.47 (14,570)	39.53 (9,524)	
College and above	72.09 (10,776)	27.91 (4,173)	
Parenting, %(n)			0.000
Others	61.16 (4,976)	38.84 (3,160)	
Parents	65.85 (20,473)	34.15 (10,615)	
Basic health knowledge of parenting, %(n)			0.000
Bad	61.80 (11,388)	38.20 (7,040)	
Good	67.19 (13,251)	32.81 (6,470)	
Area			0.000
Western China	66.97 (9,779)	33.03 (4,822)	
Middle China	59.39 (6,479)	40.61 (4,430)	
Eastern China	67.02 (9,191)	32.98 (4,523)	
Total	64.88 (25,449)	35.12 (13,775)	

### Multivariate results

Results of multilevel logistic regression predicting antibiotic misuse are showed in Table 3. After controlling potential confounders and interactions, multilevel logistic regression revealed that urban area (OR = 0.79 (0.76, 0.83)), female children (OR = 0.92 (0.88, 0.96)), higher education of guardians (OR = 0.60 (0.55, 0.66)), being raised by parents (OR = 0.90 (0.85, 0.94)), guardians having basic health knowledge (OR = 0.82 (0.79, 0.86)) were protective factors and children’s age (1–3 years OR = 1.62 (1.54, 1.71);4–6 years OR = 1.90 (1.77, 2.03)) was risk factor of antibiotic misuse among children 0–6 years with diarrhea in China.

**Table 3:** Results of multilevel logistic regression predicting antibiotics misuse among children with diarrhea in China.

Independent variables	Western China OR (95% CI)	P value	Middle China OR (95% CI)	P value	Eastern China OR (95% CI)	P value	Total OR (95% CI)	P value
Place of residence (urban)	0.69 (0.64, 0.74)	0.000	0.67 (0.60, 0.74)	0.000	1.06 (0.97, 1.17)*	0.216	0.79 (0.76, 0.83)	0.000
Children’s gender (female)	0.91 (0.85, 0.98)	0.015	0.93 (0.86, 1.01)*	0.084	0.92 (0.85, 0.99)	0.028	0.92 (0.88, 0.96)	0.000
Children’s age								
1–3 years	1.63 (1.51, 1.78)	0.000	1.48 (1.33, 1.65)	0.000	1.85 (1.68, 2.05)	0.000	1.62 (1.54, 1.71)	0.000
4–6 years	1.71 (1.50, 1.94)	0.000	1.99 (1.75, 2.26)	0.000	2.15 (1.91, 2.45)	0.000	1.90 (1.77, 2.03)	0.000
Household income per capita^a^								
Middle	1.63 (1.51, 1.78)	0.000	0.86 (0.77, 0.96)	0.006	0.96 (0.86, 1.07)*	0.503	1.02 (0.96, 1.08)*	0.560
High	1.71 (1.50, 1.94)	0.027	0.98 (0.85, 1.14)*	0.838	0.75 (0.65, 0.86)	0.000	0.97 (0.89, 1.05)*	0.410
Education of guardians (college and above)^a^	0.54 (0.46, 0.63)	0.000	0.73 (0.60, 0.88)	0.001	0.65 (0.56, 0.76)	0.000	0.60 (0.55, 0.66)	0.000
Parenting (parents)	1.05 (0.96, 1.15)*	0.246	0.86 (0.79, 0.95)	0.002	0.87 (0.79, 0.95)	0.003	0.90 (0.85, 0.94)	0.000
Basic health knowledge of parenting (good)	0.92 (0.85, 0.98)	0.016	0.93 (0.86, 1.01)*	0.090	0.66 (0.61, 0.71)	0.000	0.82 (0.79, 0.86)	0.000

**Notes:**

* Non-significant (P > 0.05).

a The interaction of education and income was adjusted.

When stratifying by areas, the results were slightly different. Place of residence, **in Eastern China,** was not associated with antibiotics use. Children gender, **in Middle China**, was not associated with antibiotics use. The guardians of middle and higher income were more likely to use antibiotics to treat infant diarrhea **in Western China**, while in **Middle China** and **Eastern China**, middle and higher income were the protective factors. Having basic health knowledge and living in **Middle China**, was not associated with antibiotics use.

## Discussion

The biological systems and organs of children are not well-developed, especially those of younger children, which make children more vulnerable. A high rate of self-medication use and particularly antibiotic abuse, might not only impede children’s development, but also lead to serious problems for clinical treatment such as drug resistance, unbalanced bacteria distribution and a variety of toxic and other side effects (Pavydė et al., 2015; Shehadeh et al., 2012).

This study revealed important findings, related to inappropriate public practice of antibiotic use in children aged 0–6 who had the disease of diarrhea. We aimed to describe the characteristics of the antibiotic misuse in the treatment of children with diarrhea and provide the basis for the government’s relevant interventions in the future.

Among the children with diarrhea, 35.12% of them were given antibiotics without prescription. The proportion even increased to 40% or above in the suburbs, children aged 4–6 years and middle China. This finding is similar to the results of previous studies in China, which found that 36% of the parents were in an urban area (Bi, Tong & Parton, 2000). In another study, 34.5% of the children with diarrhea were given antibiotics by their parents (Bi, Tong & Parton, 2000), in contrast to 12% in the suburbs and 23% in the urban areas of Greece (Edwards et al., 2002; Mitsi et al., 2005).

In our study, parents in suburbs are more likely to misuse antibiotics, probably as a result of lower access to health information in these areas. This is in accordance with the findings of other researchers, who found that the frequency of dispensing antibiotics without a medical prescription and the frequency of prescribing antibiotics for children and the elderly are significantly higher in lower socio-economic areas (Farah et al., 2015; Grigoryan et al., 2006). The process of urbanization in eastern regions is more advanced than that in other regions and the selected organizations are consistent with the socio-economic level, which may explain the results in our study.

The prevalence of antibiotic use in boys is higher than that in girls in Western and Eastern China. A study on antibiotic body burden in the Chinese children attending school also showed a difference in gender. They inferred that may be related to the gender-related differences in dietary habit or lifestyle, which influenced the exposure risk of children to these antibiotics from the environment or food (Wang et al., 2015). Another possible reason may be that boys are more valued or cared than girls and so boys are more likely to be asked to take antibiotics early (Yount, 2003).

As the age of children increases, the proportion of guardians using antibiotics for their children is getting higher. The most episodes of diarrhea are self-limiting and typically last 3–4 days. Guardians may develop incorrect perceptions of the efficacy of some treatments. That is, guardians may assume that the diarrhea stopped because of treatments being given in time. Guardians may try more energetically to head off the diarrhea episode before it progresses and at occasions what they know to be a costly set of treatments (Zwisler, Simpson & Moodley, 2013). Since parents often lack the patience to wait for natural rehabilitation of their children, they may have the illusion that antibiotics can decrease the duration or minimize the complications of diseases (Nyquist et al., 1998; Rousounidis et al., 2011).

In an overall view, neither middle income nor high income has significant influence on antibiotic misuse among children. When disaggregated by region, both of them are risk factors in western region while middle income in middle China and high income in the eastern China are protective factors. Family income was only documented in a very few surveys on antibiotic use and the association between family income and antibiotic use (You et al., 2008). According to previous studies in Jordan, self-medication prevails as income increases (Al-Azzam et al., 2007; Sawair et al., 2009).

The lower the degree of education of the children’s guardians, the more easily for them to abuse antibiotics, different regions showed the same trend. Lower education level/family income and the male gender were identified as predictors of poor knowledge and inappropriate behavior for antibiotic use, respectively (You et al., 2008). However, the association between education level and antibiotic misuse is complicated. Evidence also suggests that well-educated mothers prefer to use medicine for their children themselves without visiting doctors, because they are more confident on their medical knowledge (Bi, Tong & Parton, 2000). Besides, in a current study, grandparents are more likely to use antibiotics than parents in **middle** and **eastern** China. This may be interpreted by the fact that grandparents hold a belief that they are qualified only if they can keep the children healthy or help them recover quickly. Study suggests that children need appropriate parental and professional support in taking control of their medication and treatment (Costello, Wong & Nunn, 2004).

Our governments should further intensify health education, to ensure the public recognize the dangers of self-treatment and unauthorized use of antibiotics. The campaign needs to be restructured by user characteristics (Kim, Moon & Kim, 2011). Carefully structured national campaigns can be successful. Educational materials that include cartoons and illustrations may be more successful than text-only materials to engage children and their parents (Andrews et al., 2012). These measures can improve public education and compliance, and drug utilization review and continuing medical education for both primary care physicians and specialists (Gilberg et al., 2003).

The relative strengths and weaknesses must be taken into account when interpreting the findings of our study. One major limitation is the cross-sectional study design. The nature of the study design does not allow for determination of causality. Longitudinal studies would be needed to confirm the relationship between antibiotic misuse and its determinants and identify the most valuable interventions. Second, our findings rely on antibiotics related infantile diarrhea rather than more comprehensive antibiotic lists, which would underestimate the prevalence of antibiotic misuse and hinder the power to reveal significant relationships. Third, our study is vulnerable to recall bias. However, children are often the main focus of most families in modern China because of the “one-child” policy and the guardians pay much attention on the health of their children. Therefore, the recall bias would make little difference on the results. Finally, parents who give antibiotics frequently to their children may prefer not to answer the questionnaire because they thought they were right, which could result in selection bias.

Our study is strengthened by the high participation rate and use of a representative sample of children and its guardians among China. Our methodology using self-answered questionnaires increases the precision of information obtained (Rousounidis et al., 2011). Besides, the national huge sample size and multi-level analyses strategy used in this study guarantee comprehensive investigation and robust results.

## Conclusions

Antibiotic misuse is a national concern. In this survey, associations between self-medication and its risk predictors, including the education level of guardian, children’s age and gender, were revealed. Minimizing antibiotics on a national scale can reduce resistance rates. The government should raise the awareness about antibiotic misuse among the public to a greater extent.

## Supplemental Information

10.7717/peerj.2668/supp-1 Supplemental Information 1 Raw data collected through the paper questionnaire answered by the guardians of the children aged 0–6 years living in the sampled communities.

## Additional Information and Declarations

### Data Deposition

The following information was supplied regarding data availability: The raw data has been supplied as Supplemental Dataset Files.
